# Primary Crystal Orientation of the Thin-Walled Area of Single-Crystalline Turbine Blade Airfoils

**DOI:** 10.3390/ma12172699

**Published:** 2019-08-23

**Authors:** Włodzimierz Bogdanowicz, Jacek Krawczyk, Robert Paszkowski, Jan Sieniawski

**Affiliations:** 1 Institute of Materials Science, University of Silesia in Katowice, 1a 75 Pułku Piechoty St., 41-500 Chorzów, Poland; 2 Department of Materials Science, Rzeszów University of Technology, 2 Wincentego Pola St., 35-959 Rzeszów, Poland

**Keywords:** turbine blade, single-crystalline superalloy, thin-walled areas, crystal orientation, dendritic growth, dendrite bending

## Abstract

The thin-walled airfoil areas of as-cast single-crystalline turbine blades made of CMSX-4 superalloy were studied. The blades were produced by the industrial Bridgman technique at withdrawal rates of 2, 3 and 4 mm/min. The angle between the [001] crystallographic direction and blade axis, related to the primary orientation, was defined by the Ω-scan X-ray diffraction method at points on the camber line located near the tip of an airfoil and at points of a line located in parallel and near the trailing edge. Additionally, primary crystal orientation was determined by Laue diffraction at the selected points of an airfoil. The influence of mould wall inclination on the primary crystal orientation of the thin-walled areas is discussed. The effect of change in the [001] crystallographic direction, named as “force directing”, was considered with regard to the arrangement of primary dendrite arms in relation to the trailing edge and the camber line. It was stated that when the distance between the mould walls is less than the critical value of about 1.5 mm the “force directing” increases as the distance between the walls of the mould decreases. The effect may be controlled by selecting an appropriate secondary orientation using a seed crystal in the blade production process. The model of dendrite interaction with the mould walls, including bending and “deflection”, was proposed.

## 1. Introduction

Single-crystalline superalloys are a group of materials characterized by increased strength properties, especially creep resistance at high operating temperatures. The strength properties of relevant grades of superalloy, such as CMSX of Cannon Muskegon and Rene of General Electric, PWA of Pratt and Whitney or RR of Rolls Royce, are successively increased in subsequent generations. For example, the operating temperature has been increased from about 1060 °C for Rene (first-generation) up to 1150 °C for TMS238 (sixth-generation). The creep rupture lives of the representative single-crystal superalloys have been lengthened from about 250 h at 1050 °C/150 MPa for a typical first-generation alloy, such as SRR99, to about 1000 h for the third-generation alloy, RR3000. The ultimate tensile strength UTS for TMS238 has been increased to 1348 MPa at 750 °C [1,2,3,4]. The crystal orientation, which depends on the parameters of directional crystallization and the geometry of the cast [5,6,7], have an important effect on the mechanical properties of single-crystalline superalloy casts [1,8,9], such as stress rupture life and elongation [10]. Additionally, the tensile strength and creep resistance of the blades depend on the presence of low-angle boundaries [11] and the localisation of low-angle boundaries is related to the cast geometry. Additionally, the residual stress in a sub-surface area that may be created during crystallization [12] may be related to the cast geometry. In thin-walled areas the volume fraction of the regions where the stress occurs is higher than in the thick blade parts, which contributes to local changes in the crystal orientation and morphology of the γʹ phase created after subsequent heat treatment. This inhomogeneity may lead to faster degradation of thin-walled areas during operation. 

It is known that the complex shape of the cast may cause local changes in crystal orientation of single-crystalline turbine blades [13] and, as may be deduced, this applies to blades’ airfoils, the complex shape of which is defined by two spirally twisted surfaces—the pressure surface and suction surface. 

The local changes in crystal orientation are related to local changes in the direction of dendrite growth, while it is commonly assumed for alloys with an fcc structure that dendrites grow strictly along the [001]-type crystallographic direction [14], which is crystallographically determined. The interaction of dendrites with tilted mould walls may cause a deviation from the preferred crystal orientation during crystallization [15]. Even small local differences (dozens of arc minutes) in the local crystal orientation cause the creation the low-angle boundaries (LAB) during casting, or later, during heat treatment, reducing the strength [11]. This effect depends on the values of the LAB misorientation angle [11,16]. In thin-walled areas of an airfoil the volume fraction of residual sub-surface stresses is high, unlike the thick root part, and subgrain boundaries pass through the entire cast—from the suction surface to the pressure surface [17]. Therefore, it is important to study the distribution of the crystal orientation in thin-walled areas of turbine blade airfoils.

**Figure 1:**
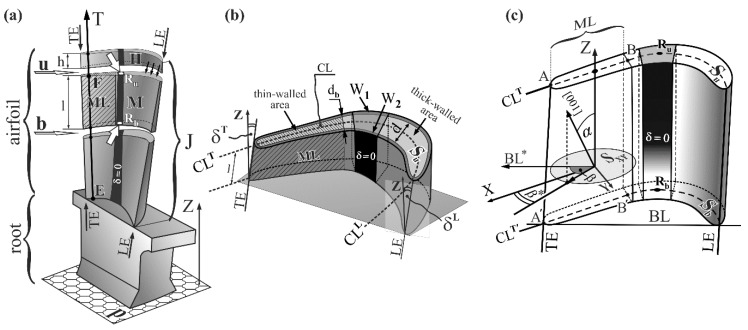
(**a**) Illustration of turbine blade with cutting scheme (LE—leading edge, TE—trailing edge, T axis is parallel to TE); (**b**) shape of sample M with scheme of mould walls arrangement (CL—camber line, S_u_—micro-section surface, W_1_, W_2_—mould walls, d—distance between mould walls, δ^T^—angle between axis Z and TE, δ^L^—angle between axis Z and LE); (**c**) geometric description of α, β and β* angles; plane S_XY_ is parallel to plane p in Figure 1a and BL* is parallel to BL.

The primary crystal orientation is mostly described by the α angle which is the angle between the [001] crystallographic direction and the Z axis of the blade (Figure 1), specifying the direction of centrifugal force during operation. Most often the Z axis, aligned to the highest load in the blades, is parallel to the withdrawal direction in the Bridgman crystallization process, related to the <001> crystallographic direction [18,19]. The α angle is one of the components defining the primary dendrite arms’ (PA) orientation, while it is commonly assumed that dendrites grow strictly along the [001] direction [20]. The orientation of the PA have an effect on the structural perfection of single-crystalline blades and, on the other hand, it may be affected by the mould walls’ inclination [21].

The inclination of mould walls in relation to the Z axis, which may be described by δ^T^ and δ^L^ angles (Figure 1b), is different for particular areas of an airfoil. Additionally, the distance *d* between the mould walls, defining the thickness of an airfoil for each lateral section, varies continuously along the camber line (CL) between the leading edge (LE, Figure 1b) and the trailing edge (TE, Figure 1b). The CL is a line equidistant from the suction surface and pressure surface of an airfoil. Therefore, it is logical that the blade airfoil may be divided into a thin-walled area and thick-walled area (Figure 1b). Considering the above, it can be assumed that the α angle, measured at points of the CL, may be different for thin-walled and thick-walled areas. This could significantly affect the direction of the dendrites’ growth and their crystal orientation in these two areas. The importance of this issue is additionally related to the fact that the tip area of the blade airfoil near the TE is critical due to the possibility of easier degradation during operation. This area is subjected, among others, to the largest vibration amplitudes [22]. The morphology and the size of the dendrites, as well as their primary spacing, depend on the crystallization rate [1,23,24] and, thus, the interaction of dendrites of different sizes with the mould walls may be dissimilar. Therefore, the blades obtained at different withdrawal rate are examined in the present study. 

The aim of the study is to determine a distribution of the α angle describing the primary crystal orientation of thin-walled areas of as-cast single-crystalline turbine blade airfoils, and to analyse the relation between this distribution and the slope of mould walls in relation to the axis of blades obtained at different withdrawal rates. An additional aim is to consider the effect of the mould walls’ inclination on the growth direction of the primary dendrite arms.

## 2. Material and Methods 

The single-crystalline blades were prepared at the Research and Development Laboratory for Aerospace Materials at the Rzeszów University of Technology, Rzeszów, Poland. The blades were solidified at withdrawal rates of 2, 3 and 4 mm/min. in an ALD Vacuum Technologies Inc. vacuum (Hanau, Germany). A Bridgman furnace using a spiral selector [25]. A withdrawal rate of 3 mm/min is an optimal rate for obtaining casts in industrial Bridgman method with best strength parameters and the other two rates are the closest rates, chosen for comparison of analysed parameters [26]. The commercial nickel-base superalloy of CMSX-4 was used. Preparation and studies of the samples were carried out in three steps. In the first step, the narrow fragment H of an airfoil tip of *h* = 5 mm was cut along the *u* plane which is parallel to the plane p and perpendicular to the blade axis Z that is opposite to the withdrawal direction (Figure 1a). The H fragment was not analysed due to its high casting strain. In the second step, the α angle distribution along the T axis which is parallel to the TE (Figure 1a) was defined using X-ray Freiberg Instruments EFG diffractometer (Freiberg Instruments, Freiberg, Germany) [27]. The values of α angle were measured between E and F points. The T axis and the TE are 1 mm apart. In the third step, the sample M (Figure 1a) was obtained by cutting the airfoils of all blades along the plane *b* (Figure 1a) parallel to the base plane p (Figure 1a). The distance *l* between the plane *b* and the plane *u* is 11 mm. The overall blade airfoil length *J* is 33 mm. 

The upper surface of M samples marked in Figure 1a by downward arrows was prepared by a careful mechanical polishing. Additionally, the upper surface of the M sample is presented in Figure 1b as a micro-section surface S_u_. The Laue diffraction patterns were obtained from R_b_ and R_u_ points of the samples M, marked in Figure 1a by tick white arrows. The points were selected in the middle fragment of the CL which is located in the region where the mould walls are parallel to the Z axis. The region is marked in black in Figure 1a and its fragment is marked as δ = 0 in Figure 1b,c, where δ is the angle of mould wall inclination relative to the Z axis. The surface area, covered by X-ray beam of Cu radiation, was about 1 mm in diameter. The Laue patterns were recorded in back-reflection geometry on the image plates (IP) using X-ray diffractometer of XRT-100 system provided by EFG Freiberg Instruments. QLaue software (version 2.0) was used for measuring the angles between the Laue reflections of the patterns and measuring the rotation component of diffraction planes relative to reference axes Z and BL (Figure 1c) as well as for indexing the reflexes. The Laue patterns were also used to determine the α and β angles (Figure 1c) defining the alignment of the [001] direction relative to the Z and BL* axes (Figure 1c). The average error of angles measurement was 0.2°. The β is the angle between projection of the [001] direction onto the plane S_XY_ (S_XY_ is parallel to the p and S_b_ planes, the X axis is perpendicular to the Y axis, Figure 1a) and the BL axis. Additionally, the Laue patterns were used to determine β* that is the angle between the projection of the [001] on the S_XY_ and the X axis (Figure 1a,c) which is parallel to the CL^T^ and CL^T^’. The [001] vector is a unit-vector used for the description of the [001] crystallographic direction only.

Considering the distance *d* measured between mould walls on the transverse micro-section surface S_u_ (Figure 1b), it was stated that for the analysed blades such distance in the fragment ML of the sample M (Figure 1a,b) is approximately constant, that is *d_b_* = 1.8 mm, and it is lower than in the other areas of the airfoil cross-section. Therefore, the airfoil may be divided into two parts: the thin-walled area ML of thickness *d_b_* (Figure 1b) and the thick-walled area which is the remaining part of the airfoil. The thin-walled area with a constant distance *d_b_* was located near the TE. In this area the CL fragment is almost rectilinear, therefore, it was marked as CL^T^ (Figure 1b), while the CL fragment, directly adjacent to the LE, was marked as CL^L^.

The complex shape of the blades makes it necessary to take into account the inclination angle of the LE and TE defined by the δ^L^ and δ^T^ angles (Figure 1b). The value of δ^L^ measured at the level of the *b* and *u* planes of the M samples is constant and is 12°. The value of δ^T^ measured at the same levels is constant too and is 1.5°. The CL is rectilinear and the δ^T^ angle is constant in the ML area. Therefore, the ML area can be represented as an area with approximately parallel side walls, distanced by the constant value *d* equal to *d_b_* (Figure 1b) and inclined at the constant angle δ^T^ relative to the Z axis. 

The α angle (Figure 1c) was determined point by point along the camber line applying the Ω-scan method with the use of an X-ray Freiberg Instruments EFG diffractometer [28]. An X-ray tube with a Cu anode was used. The surface area covered by X-ray beam was 0.7 mm in diameter. The average measurements error was 0.02°. 

A JEOL JMS-6480 scanning electron microscope (JEOL Ltd., Tokyo, Japan) was used to visualize the dendritic structure. The macro-SEM images of the fragment of the S_u_ surface corresponding to the ML area (Figure 1c) were created by stitching of separate smaller SEM micrographs. 

## 3. Results and Discussion

**Figure 2:**
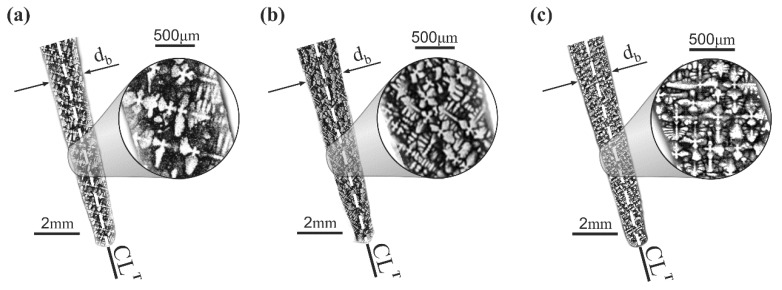
Exemplary dendritic structures of ML thin-walled airfoil area of blades produced at withdrawal rate of (**a**) 2 mm/min.; (**b**) 3 mm/min.; and (**c**) 4 mm/min. (SEM, BSE technique).

The macro-SEM images obtained from a fragment of the S_u_ surface that corresponds to the thin-walled area ML (Figure 1a) show a typical dendritic structure (Figure 2).

**Figure 3:**
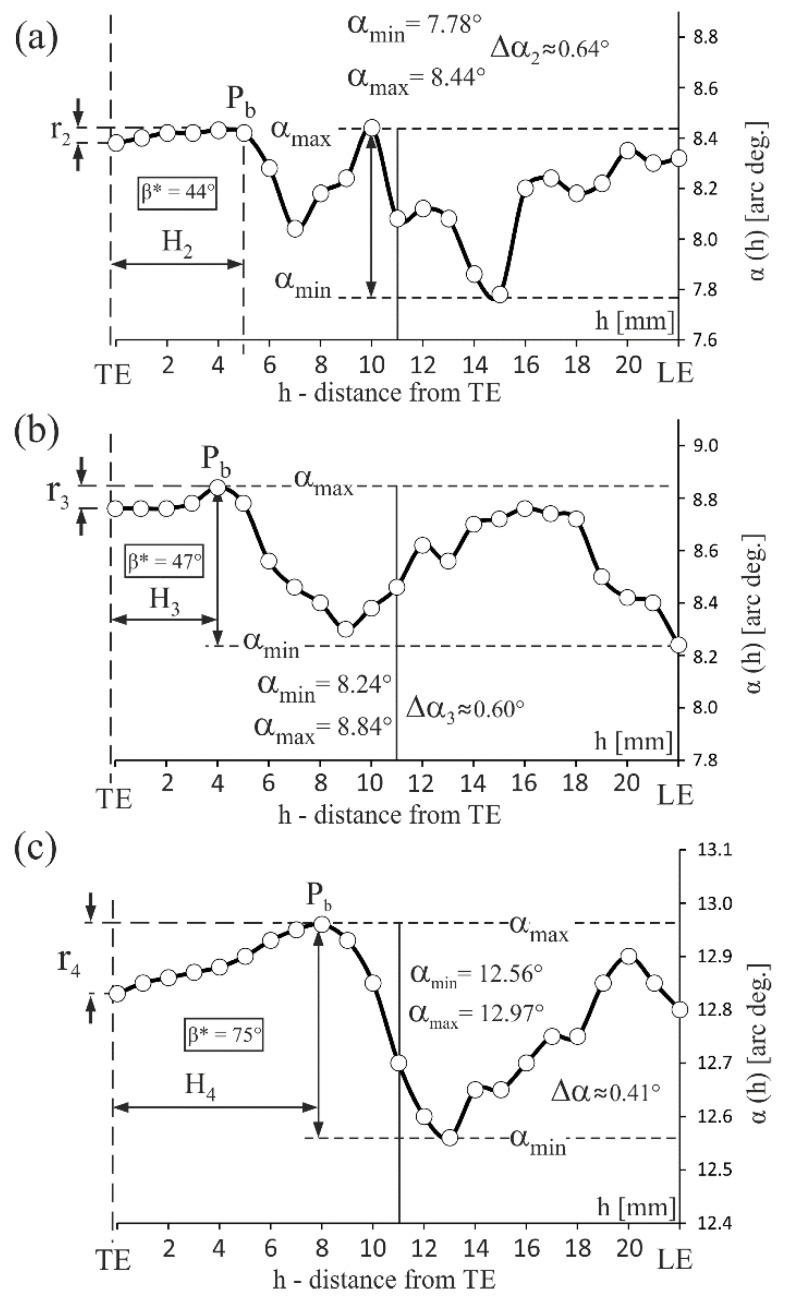
Examples of the α angle distribution along the camber line of the S_u_ surface for blades obtained at the withdrawal rate of (**a**) 2 mm/min.; (**b**) 3 mm/min. and (**c**) 4 mm/min.

The α angle distribution along the CL of the S_u_ surface, i.e., α(h) relationship, where *h* is the distance from the TE, for the blades obtained at withdrawal rates of 2 mm/min., 3 mm/min. and 4 mm/min. are presented in Figure 3. The analysis of the α(h) relationship allows us to conclude that there are fluctuations Δα in the range of changes less than 1° for all airfoils (Δα_2_ ≈ 0.64°, Δα_3_ ≈ 0.60°, Δα_4_ ≈ 0.41°). The character of α(h) near the TE is different than in the remaining parts of the CL. In the area near the TE (H_2_–H_4_ areas, Figure 3), where the crystallization takes place between the close-together walls of the mould, there were very low α angle variations (r_2_ < Δα_2_, r_3_ < Δα_3_, r_4_ < Δα_4_, Figure 3) and the α value decreased as it approached the TE. In the remaining fragment of the CL, the α(h) relationship possesses a stochastic character with large fluctuations. It can be related to the subgrain structure and low-angle boundaries [13]. The values of the α angle, defined for all analysed blades’ airfoils, ranged between 5° and 15°. 

The inclination of mould walls relative to the Z axis near the TE was lower than the α angle (δ^T^ = 1.5° < α) for all analysed samples. Considering the commonly used assumption that dendrites grow strictly towards the [001] crystallographic direction it was concluded that near the TE, in the thin-walled area ML of an airfoil (Figure 1b), mould walls can force the growth direction of the primary dendrite arms during crystallization of a blade. Therefore, the range of the α changes in the thin-walled areas (H_2_–H_4_) compared to other areas is smaller (r_2_ < Δα_2_, r_3_ < Δα_3_, r_4_ < Δα_4_; Figure 3). The distance *d* between mould walls (Figure 1b) may be one of the factors that contributes to the changes of α(h) character. 

The revealed effect of “force directing” by the mould walls may not occur when the primary arms (PA) of dendrites grow parallel to the walls. Assuming that the [001] direction is parallel to the PA, it may be concluded that the force directing effect should depend upon the crystal orientation of the PA in relation to the surfaces of mould walls which are parallel to the CL^T^ and TE for the ML area (Figure 1b,c). 

**Figure 4:**
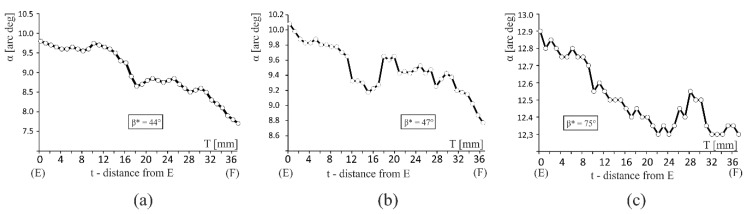
Examples of the α angle distribution along the T axis (Figure 1a) of an airfoil for blades obtained at the withdrawal rate of (**a**) 2 mm/min.; (**b**) 3 mm/min. and (**c**) 4 mm/min. t—distance from the E point—Figure 1a.

Figure 4 presents examples of the α distribution along the T axis of an airfoil. The distance *t* = 0 corresponds to the E point that is located on the plane of which the platform of the blade root is part of (Figure 1a). It can be seen that the α angle decreases with local changes from the E point (*t* = 0 mm) to the F point (*t* = 37 mm). It can be concluded that the force directing effect in the thin-walled area ML occurs, and it is progressing from the root platform towards the tip of the blade.

**Figure 5:**
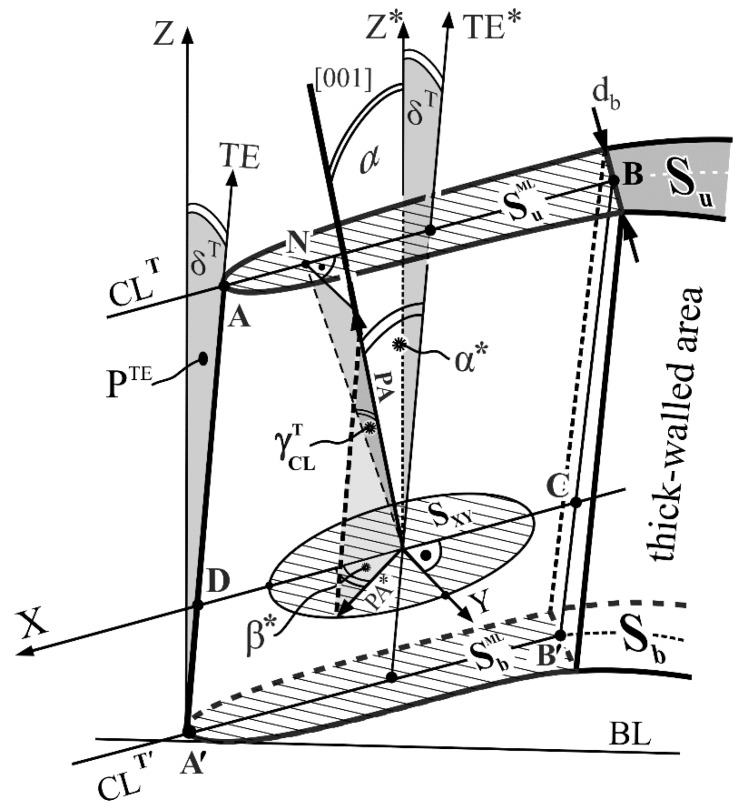
Scheme of the [001] crystallographic direction arrangement which is a primary arm (PA) arrangement in the thin-walled airfoil area ML. The PA unit-vector defines the direction of the primary arm growth. The CL^T^ is parallel to CL^T^ʹ and the X axis; Z is parallel to Z*, TE is parallel to TE*, $S_{u}^{\mathrm{ML}}$ is a fragment of Su, $S_{b}^{\mathrm{ML}}$ is a fragment of Sb; $S_{b}^{\mathrm{ML}}$ is parallel to $S_{u}^{\mathrm{ML}}$ and S_XY_.

Let’s consider in detail the geometric relationship between the inclination of lateral surfaces in an airfoil thin-walled area ML and the most important angles defining the [001] direction which is specific for the growth process of the PA. The scheme presented in Figure 5 will be helpful for analysis. The following details are important for further consideration: The Z* and Z axes in Figure 5 are perpendicular to the planes $S_{u}^{ML}$, $S_{b}^{ML}$ and S_XY_. The TE^*^ is parallel to the TE. The CL^T^ and CL^T’^ are parallel to the X axis. The X axis is perpendicular to the Y axis. The TE is inclined to the Z axis by the δ^T^ angle. The surface P^TE^ is perpendicular to the CL, CL^T^’ and X. The BL in Figure 5 is the same baseline presented in Figure 1c. The PA are presented in Figure 5 by the PA unit-vector in order to visualize their growth direction only. The angle between the PA and Z* axis is marked by the α angle. The growth direction of the PA was always taken as parallel to the [001] crystallographic direction, as it is often assumed [28,29]. Recent studies [30] show that the difference in the direction of dendrite growth correlates with difference in crystal orientation, which confirms the above assumption. Since the TE is parallel to the TE^*^, the angle between the PA and the trailing edge TE may be denoted as the α* angle (Figure 5). The fragments AB and AʹBʹ of the CL in the thin-walled area ML (Figure 1b) are parallel straight lines for the studied type of blades. In Figure 5, the N point is the orthogonal projection of the PA vector end on the ABCD plane, formed by the X axis and the TE, arranged parallel to the mould walls. $\gamma_{CL}^{T}$ is the angle between the PA and the mould wall. 

The PA orientation in relation to the X axis is represented by β* which is the angle between the X axis and the PA*. The PA* is the projection of the PA on the S_XY_ surface. The α angle values for the R_b_ and R_u_ points were determined from the Laue diffraction patterns recorded at these points. The β* values of the thin-walled area ML were determined on the basis of the Laue diffraction patterns recorded at the R_u_ point only, while the $\gamma_{CL}^{T}$ values were determined on the basis of patterns recorded at the R_b_ and R_u_ points, using a method described in the Appendix A. The values of β* are presented in Figure 3 and Figure 4.

Table 1 presents values of $\gamma_{CL}^{T}$ determined for the $S_{b}^{ML}$ and $S_{u}^{ML}$ surfaces and their differences $\Delta \gamma_{CL}^{T}$ as well as the α values determined for the R_b_ and R_u_ points of an airfoil of blades obtained at different withdrawal rates. 

Analysis of the data presented in Table 1 shows that the decrease of $\gamma_{CL}^{T}$ of about several or over a dozen degrees is observed. The decrease takes place within the range of planes $S_{b}^{ML}$ and $S_{u}^{ML}$ towards $S_{u}^{ML}$. It can be concluded that the decrease of the $\gamma_{CL}^{T}$ angle reveals that the force directing effect progresses from the $S_{b}^{ML}$ to $S_{u}^{ML}$ surface. On the other hand, the α angle values, at the points R_b_ and R_u_, are almost equal (Figure 1a). These points belong to the columnar area of the M samples, for which the inclination of the mould wall surfaces to the Z axis is zero (δ = 0) (Figure 1).

**Figure 6:**
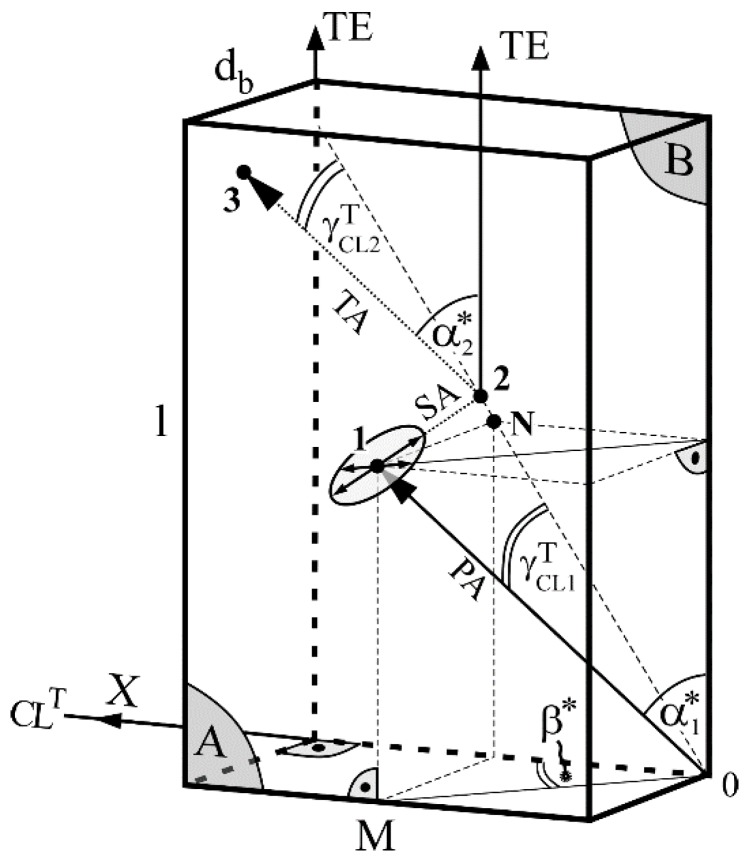
Scheme of primary arm (PA), secondary arm (SA) and tertiary arm (TA) growth in the thin-walled area of airfoil. A and B are the mould wall surfaces. The angles are enlarged for figure clarity.

The cuboidal thin-walled fragment of an airfoil marked in Figure 1a by the ML is presented in Figure 6. The influence of the mould walls on the arrangement of the [001] crystallographic direction and on the orientation of primary arm (PA), secondary arm (SA) and tertiary arm (TA) directions can be determined according to the scheme presented in Figure 6. The *d_b_* is the thickness, *l* is the height and M is the width of the ML. The vector situated between points 0 and 1 indicates the PA direction. The crossed small arrows in the grey circle indicate the directions of the secondary dendrite arms. The N point in Figure 6 is the orthogonal projection of the point 1 on the B plane. The points 1 and 3 belong to the A plane, and point 2 to the B plane. At point 1 the PA is inclined to the B and A planes at a small angle $\gamma_{CL1}^{T}$. According to the model presented in [13] the PA growth direction slightly changes near point 1, decreasing the angle between the PA and A plane. When the angle decreases, the growth of the PA is impeded and a secondary dendrite arm begins to grow along the segment 1–2. The process has a different character at point 2 where the angle between the secondary dendrite arms and the mould wall is close to 90°. In this case, the strict orthogonal growth of the tertiary dendrite arms in relation to the secondary arms occurs [13]. The further growth of the tertiary dendrite arm takes place along the segment 2–3. This process may be called a “deflection” of dendrite arms from the mould wall. During “deflections”, there is a slight deviation of the PA growth direction near point 1 and the tertiary arms’ (TA) growth direction near point 3 [13]. The mechanism of the deviation is related to the asymmetry of the diffusion area, i.e., the asymmetry distribution of solute in liquid ahead of the PA near mould wall A. At the points 1 and 3, the asymmetry of the diffusion area manages to form, whereas at point 2, the asymmetry of the diffusion area does not manage to form, due to the high angle (close to 90°) between the secondary dendrite arms and the mould wall. The reasons of the “morphological bending” proposed in [30,31] may be similar to the reasons of the [001] crystallographic direction deviation in thin-walled areas. The critical angle may exist between dendrites and a mould wall. If the angle is higher than critical, the strictly orthogonal “deflection” of dendrites takes place, otherwise, the dendrites bend.

The “deflections” of the dendrites at the 1- and 3-type points causes progressive inclination of subsequent PA- and TA-type dendrites’ arms parallel to the mould walls’ planes (planes A and B). The angle between the mould walls and the [001] crystallographic direction, which is the direction of subsequent dendrite arms growing roughly parallel to the mould walls, is reduced ($\gamma_{CL2}^{T}\;$< $\gamma_{CL1}^{T}$, Figure 6). The $\gamma_{CL2}^{T}$ and $\gamma_{CL1}^{T}$ are the angles directly related to the α angles. Due to the fact that the angle δ^T^ of the mould walls’ inclination is low (1.5°) and the α angle values for the analysed blades are higher (of about several or over a dozen degrees, Table 1), the force directing effect causes a gradual decrease in the α angle when approaching the airfoil tip in the thin-walled area (Figure 4). Otherwise, the force directing effect may increase the α angle. Similar “deflections” processes are described in [30] as “re-emerging”. The authors suggest that the “re-emerging” process may reduce the degree of mosaicity because the short dendrites in the thin-walled areas bend less than the long dendrites in the thick-walled areas due to geometrical constraints [30]. The description of mosaicity creation in the thin-walled area should include not only the length of dendrites, but also the angle between the stems of dendrites and a mould wall. The two parameters should be taken into consideration: the critical length of dendrite [30] and the critical angle between the stems of dendrites and a mould wall. 

Near the TE, the force directing effect is expressed in the graphs in Figure 3 by the monotonic decrease of the α angle in the H_2_, H_3_, H_4_ areas when approaching the TE. The α angle starts to decrease at point P_b_. The thickness of the airfoil at point P_b_ is about 1.5 mm. The deviation of dendrites at each of 1- and 3-type points (Figure 6) is small, however, the total reduction of the $\gamma_{CL}^{T}$ angle caused by the “deflection” in a series of points may be significant. The aforementioned reduction ${\Delta \gamma }_{CL}^{T}$ for withdrawal rates of 2, 3 and 4 mm/min. was 4.5°, 11.5° and 16.5°, respectively (Table 1). The ${\Delta \gamma }_{CL}^{T}$ value may depend on the number of reflections at the 1- and 3-type points (Figure 6) and, in turn, the number of reflections is higher for larger β*. This is confirmed by the fact that, for blades obtained at withdrawal rate of 4 mm/min., with maximal β* = 75° (Figure 3), the ${\Delta \gamma }_{CL}^{T}$ value is the highest (Table 1). Additionally, the H_4_ and r_4_ parameters of the α(h) relationship (Figure 3) have the highest values for these blades. The force directing effect is also expressed by reduced variation of α near the TE in the H_2_, H_3_, and H_4_ areas (Figure 3) in comparison to other fragments of the CL (r_2_ < Δα_2_, r_3_ < Δα_3_, r_4_ < Δα_4_; Figure 3). The number of “deflections” are related to α* which is the angle between the [001] crystallographic direction and the TE. There is a higher number of “deflection” processes for the higher values of the α* angle, therefore, the force directing effect is stronger. The number of deflections depends on the primary and secondary dendrite arms’ orientation in relation to the mould walls.

The force directing effect depends on at least two parameters, the distance *d* between the mould walls and the inclination angle of the PA in relation to the mould surface. The force directing effect may occur if the above-mentioned distance *d* and angle are both small. Both of these conditions are met in the thin-walled ML area. In the thick-walled area for δ = 0 (Figure 1), the inclination angle is small, but the distance between the walls of the mould is large, therefore, the force directing effect is not observed. According to the model presented above, the more 1- and 3-type points appear, the stronger the force directing effect, which is related with more dendrite “deflection” processes and, thus, the higher height *l* of an airfoil thin-walled area. Such processes will occur only if the $\gamma_{CL}^{T}$ angle is not higher than a certain limit angle, beyond which the dendrite “deflection” (bend) will not occur, which impedes the growth of the dendrite and generation of strictly orthogonal subsequent dendrite arms will occur, as in the case of point 2 (Figure 6). The analysis of the data presented in [32] shows that the value of the critical angle varies in the range of 20°–22°. Calculations of this angle should be based on the analysis of the asymmetry’s shape of the solute distribution in the diffusion area ahead the dendrite’s tip near a mould wall. The asymmetry should also depend on the withdrawal rate. The assessment of the benefits of the force directing effect causing the possible reduction of the α angle is not unambiguous. The reason for that is the possible existence of casting strains [13,33,34] near the 1- and 3-type points (Figure 6), which, after heat treatment, can cause the formation of low-angle boundaries (LAB). Such LABs cross the entire thin-walled area of an airfoil. 

Near the mould walls of the thin-walled areas, an additional mechanism of the change in the dendrite growth direction may occur. The mechanism is based on the fact that near the mould walls, the isotherms and the crystallization front can be curved [35,36], and this, considering the small distance between the mould walls in the thin-walled areas, may cause a noticeable change in the dendritic growth direction. 

## 4. Conclusions

In airfoils’ thin-walled areas of single-crystalline blades made of CMSX-4 by the Bridgman technique at withdrawal rates of 2, 3 and 4 mm/min., the inclination of the mould walls to the blade axis changes the arrangement of the [001] crystallographic direction in relation to the blade axis. It may cause a decrease of the α angle between the [001] crystallographic direction and the Z axis of a blade. This effect, called “force directing”, occurs when the distance between the mould walls is less than the critical value of about 1.5 mm. The effect generally depends on the arrangement of the [001] crystallographic direction in relation to the camber line and in relation to the trailing edge, and the effect may be explained by the influence of the mould walls on the dendrite arms’ growth direction. The “force directing” may positively affect the strength of thin-walled airfoil areas due to reduction of the α value and its variations. The effect may be controlled by selecting the appropriate secondary orientation using a seed crystal in the blade production process. To determine the optimal secondary orientation it is necessary to create a more detailed model of dendrites’ “deflections”. This is very important for slim, cored airfoils of blades.

## Figures and Tables

**Table 1:** Values of $\gamma_{CL}^{T}$, determined for the $S_{b}^{ML}$ and $S_{u}^{MM}$ surfaces, and their differences $\Delta \gamma_{CL}^{T}$, as well as the α value, determined for the R_b_ and R_u_ points of an airfoil of blades obtained at different withdrawal rates.

Parameter	Withdrawal Rate [mm/min.]					
	2		3		4	
$S_{b}^{ML}/S_{u}^{ML}$	$S_{b}^{ML}$	$S_{u}^{ML}$	$S_{b}^{ML}$	$S_{u}^{ML}$	$S_{b}^{ML}$	$S_{u}^{ML}$
$\gamma_{CL}^{T}$	9.5	5.0	12.0	0.5	18.0	1.5
$\Delta \gamma_{CL}^{T}$	4.5		11.5		16.5	
$\alpha_{R_{b}}/\alpha_{R_{u}}$	$\alpha_{R_{b}}$	$\alpha_{R_{u}}$	$\alpha_{R_{b}}$	$\alpha_{R_{u}}$	$\alpha_{R_{b}}$	$\alpha_{R_{u}}$
	8.4	8.5	9.0	9.2	13.0	13.0

**Figure A1:**
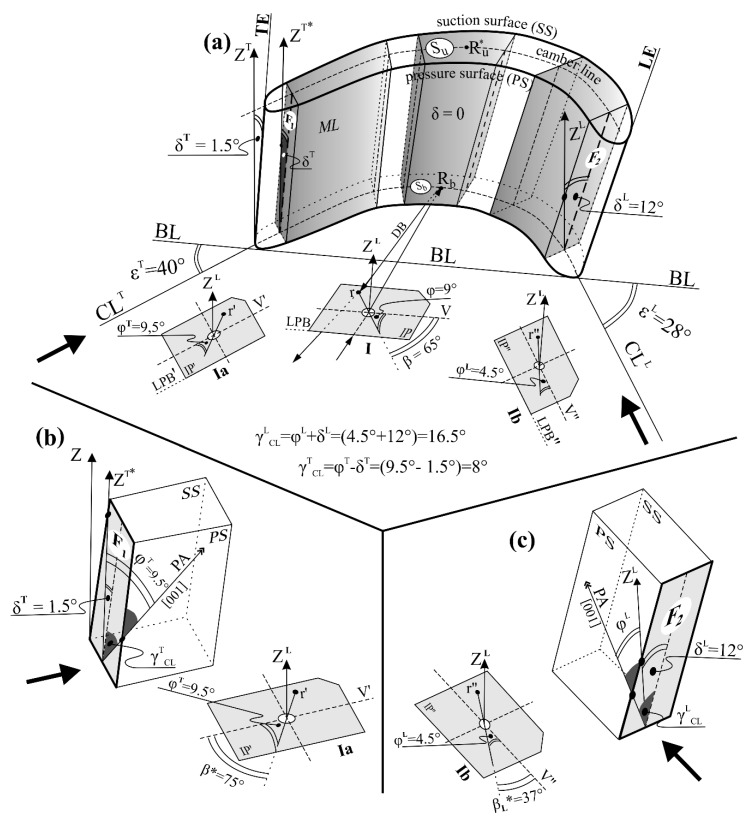
(**a**) Illustration of sample M with marked R_b_ and R_u_ points at which the Laue diffraction patterns were recorded, the image plate (IP) location and position r of the reflection from (001)-type diffraction planes in the initial Laue pattern I on the IP, as well as a hypothetical Laue pattern obtained by IP rotation relative to Z^L^ with marked new positions r’ and r’’ of the same reflection on the rotated Laue patterns Ia and Ib; (**b**,**c**) diagrams for calculating the angles between the [001] direction parallel to the primary dendrite arms and surfaces of the mould. F_1_ and F_2_ surfaces of M sample section were perpendicular to the CL^T^ and CL^L^ fragments of CL and parallel to the Z axis. Large black arrows indicate the view direction. TE: trailing edge; LE; leading edge.

## Appendix A

A method to determine the angle between the [001] crystallographic direction and the mould walls is presented below. As an example, the calculation of the angles for the area of an airfoil located both near the trailing edge TE (for ML area) and near the leading edge LE are presented.

The calculation of the γ^T^_CL_ (Figure 5) and γ^L^_CL_ angles for S_b_ plane were performed on the basis of one Laue pattern obtained on an image plate (IP) from the R_b_ point of the M sample (Figure 1a,c and Figure A1). The calculation of the γ^T^_CL_ and γ^L^_CL_ angles for S_u_ plane, performed on the basis of Laue pattern obtained from the R_u_ point, is similar. It was assumed that the trailing edge (TE) and the leading edge (LE) for the M sample are segments of straight lines inclined to the Z axis (Z^T^* is parallel to the Z^T^ and Z^L^) at the angles of δ^T^ = 1.5° and δ^L^ = 12°. The Laue pattern baseline (LPB), i.e., the baseline of the IP, was parallel to the BL of the M sample (Figure 1c and Figure A1a). The Laue pattern plane, i.e., the plane of the IP, was perpendicular to the Z axis, and the Laue pattern axis Z^L^ was parallel to the Z axis. The Laue pattern was obtained in reflective geometry. Therefore, the diffraction beam DB is directed approximately opposite the path of the PA growth.

Based on the shape of the micro-section S_u_ (Figure 1b and Figure A1a), the angles between the CL and BL for the ML area near the TE-ε^T^_,_ and near the LE-ε^L^, were determined. The ε^T^ is 40° and the ε^L^ is 28°. Afterwards, using the QLaue software (version 0.2), the Laue patterns recorded at the point R_b_ were rotated around the Z^L^ by the angle ε^T^ and ε^L^, until the LPB takes the position parallel to the CL^T^ and CL^L^. The reflex obtained from the (001)-type diffraction planes marked as r on the initial Laue pattern I, changed location to r’ and r’’ on the rotated Laue patterns Ia and Ib, respectively (Figure A1a). Based on the rotated Laue patterns, the angles ϕ^T^ and ϕ^L^ of rotation of the (001)-type diffraction planes relative to the V’ and V’’ axes (Figure A1b,c) were determined by QLaue software. In the cubic system the [001]-type crystallographic directions are perpendicular to the (001)-type planes, therefore, the ϕ^T^ and ϕ^L^ angles allow to define the inclination of the [001] crystallographic direction and the PA growth direction marked in Figure A1b,c by a double arrow in relation to the pressure surface (PS) and suction surface (SS), i.e., in relation to the mould walls. Knowing the δ^T^ and δ^L^ angles from the formulas $\gamma_{CL}^{T}$ = ϕ^T^ ± δ^T^ and $\gamma_{CL}^{L}$ = ϕ^L^ ± δ^L^, the values of $\gamma_{CL}^{L}$ and $\gamma_{CL}^{L}$ may be calculated. For example, in the case of blades obtained at the withdrawal rate of 4 mm/min., the angles ϕ^T^ = 9.5° and ϕ^L^ = 4.5°. Therefore, $\gamma_{CL}^{T}$ = ϕ^T^ – δ^T^ = 9.5° – 1.5° = 8.0°, and $\gamma_{CL}^{L}$ = ϕ^L^ + δ^L^ = 12° + 4.5° = 16.5°. Additionally, the Laue patterns Ia and Ib allow to determine the β* and $\beta_{L}^{*}$ angles that are the angles between the projection of the [001] direction on the S_b_ plane and the CL^T^ and CL^L^ (Figure A1b,c).
